# Clinical course and prognostic factors of patients in severe accidental hypothermia with circulatory instability rewarmed with veno-arterial ECMO - an observational case series study

**DOI:** 10.1186/s13049-017-0388-7

**Published:** 2017-05-02

**Authors:** Sylweriusz Kosiński, Tomasz Darocha, Anna Jarosz, Aleksander Zeliaś, Mirosław Ziętkiewicz, Paweł Podsiadło, Tomasz Sanak, Kinga Sałapa, Jacek Piątek, Janusz Konstany-Kalandyk, Robert Gałązkowski, Paweł Krawczyk, Łukasz Krzych, Rafał Drwiła

**Affiliations:** 1 Department of Anesthesiology and Intensive Care, Pulmonary Hospital, Zakopane, Poland; 2 Tatra Mountain Rescue Service, Zakopane, Poland; 3 Department of Anaesthesiology and Intensive Care, Medical University of Silesia, ul. Ziołowa 45/47, 40-635 Katowice, Ochojec Poland; 4 Polish Medical Air Rescue, Warsaw, Poland; 5 Department of Anesthesiology and Intensive Care, the John Paul II Hospital, Medical College of Jagiellonian University, Cracow, Poland; 6 Clinic of Interventional Cardiology, Institute of Cardiology, Jagiellonian University Medical College, John Paul II Hospital, Krakow, Poland; 7 Polish Society for Mountain Medicine and Rescue, Szczyrk, Poland; 8 Department of Disaster Medicine and Emergency Care, Jagiellonian University Medical College, Krakow, Poland; 9 Department of Combat Medicine, Military Institute, Warsaw, Poland; 10 Department of Bioinformatics and Telemedicine, Jagiellonian University Medical College, Cracow, Poland; 11 Department of Cardiovascular Surgery and Transplantology, Institute of Cardiology Collegium Medicum, Jagiellonian University, John Paul II Hospital, Cracow, Poland; 12 Department of Emergency Medical Services, Medical University of Warsaw, Warsaw, Poland

**Keywords:** Critical care, Extracorporeal membrane oxygenation, Hypothermia, Rewarming, Shock

## Abstract

**Background:**

Recently, veno-arterial extracorporeal membrane oxygenation (VA-ECMO) has become the rewarming treatment of choice in hypothermic cardiac arrest. The detailed indications for extracorporeal rewarming in non-arrested, severely hypothermic patients with circulatory instability have not been established yet. The primary purpose of the study was a preliminary analysis of all aspects of the treatment process, as well as initial identification of mortality risk factors within the group of severely hypothermic patients, treated with arteriovenous extracorporeal membrane oxygenation (VA-ECMO). The secondary aim of the study was to evaluate efficacy of VA-ECMO in initial 6-h period of treatment

**Methods:**

From July 2013 to June 2016, thirty one hypothermic patients were accepted for extracorporeal rewarming at Severe Accidental Hypothermia Center, Cracow. Thirteen patients were identified with circulatory instability and were enrolled in the study. The evaluation took into account patients’ condition on admission, the course of therapy, and changes in laboratory and hemodynamic parameters.

**Results:**

Nine out of 13 analyzed patients survived (69%). Patients who died were older, had lower both systolic and diastolic pressure, and had increased creatinine an potassium levels on admission. In surviving patients, arterial blood gases parameters (pH, BE, HCO_3_) and lactates would normalize more quickly. Their potassium level was lower on admission as well. The values of the core temperature on admission were comparable. Although normothermia was achieved in 92% of patients, none of them had been weaned-off VA-ECMO in the first 6 h of treatment.

**Discussion and Conclusions:**

In our preliminary study more pronounced markers of cardiocirculatory instability and organ hypoperfusion were observed in non-survivors. Future studies on indications to extracorporeal rewarming in severely hypothermic, non-arrested patients should focus on the extent of hemodynamic disturbances. Short term (<6 h) treatment in severe hypothermic, non-arrested patients seems to be not clinically appropriate.

## Background

The hypothermia-induced circulatory failure is multifactorial. In the course of cooling dehydration, fluid shifts, and increased urine output deplete the intravascular volume and cause haemoconcentration. In severe hypothermia the decrease in heart rate and rhythm disturbances result in fall in cardiac output (CO). Systemic vascular resistance falls as catecholamine release is blunted [1]. The further decrease of cardiac output may result from cardiac contractile dysfunction [2]. During and after rewarming, a hypothermia-induced cardiac failure ranging from mild reduction of CO to the fulminant circulatory shock is referred to as rewarming shock [3].

Recently, veno-arterial extracorporeal membrane oxygenation (VA-ECMO) has become the rewarming treatment of choice to be instituted in the presence of hypothermic cardiac arrest or severe circulatory instability [4–6]. One of the major advantages of VA-ECMO seems to be possibility of prolonging cardiorespiratory support if required after rewarming e.g. continuing circulatory instability, arrhythmias or impaired tolerance for the low-flow state [4, 7, 8].

Due to low availability of extracorporeal life support (ECLS) equipment, few centers worldwide undertake extracorporeal rewarming attempts in patients with accidental hypothermia. One of the few such centers is Severe Hypothermia Treatment Center (SHTC) established in Cracow, Poland. Since all stages of diagnostics and treatment have been based on the same original protocol, there is a unique opportunity to conduct an initial comparative data analysis of patients treated under the same conditions in one center in a relatively short period of time [9].

The use of ECLS for HT III patients (core temperature <28 °C, not in cardiac arrest) may be considered in the several situations [4], but the detailed indications for VA-ECMO rewarming in this group of patients have not been established yet. Thus, the primary aim of the study was a preliminary analysis of all aspects of the treatment process, as well as initial identification of mortality risk factors in patients with cardiocirculatory instability in the course of severe accidental hypothermia treated with VA-ECMO.

The secondary aim of the study was to evaluate efficacy of VA-ECMO in initial 6-h of treatment, which – at least in theory, taking into account achievable rate of rewarming, should allow for stabilization of core temperature and cardiocirculatory disturbances.

## Methods

From November 2013 to June 2016, 31 severely hypothermic patients (core temperature < 28 °C) have been rewarmed with VA-ECMO. Twelve of them fulfilled the criteria of cardiocirculatory instability (systolic blood pressure <90 mmHg and/or life-threatening arrhythmia) [4, 5]. One patient was admitted with core temperature 29,0 °C, but met all other clinical criteria of inclusion. Two patients suffered from cardiac arrest (ventricular fibrillation) during transport to the center, but in both cases circulation had been restored after short duration cardiopulmonary resuscitation (in 8th and 15th minute). Therefore, 13 patients were included in this study. Hypothermia was consecutive of urban exposure in all patients.

Vascular access was obtained via the femoral artery and vein with 22-24 Fr venous and 17-21 Fr arterial cannulas (Bioline Coating, Maquet, Rastatt Germany). The Rotaflow Console REF 706037 (Maquet, Rastatt, Germany) unit, with Heat Unit HU 35 heat exchanger and oxygenator (Permanent Life Support Set, Maquet, Rastatt, Germany) was used in all cases.

The clinical characteristics of 13 analyzed patients included: sex, age, type of exposure, type and distance of transport, vital signs on arrival (heart rhythm, core temperature, blood pressure, type of arrhythmia), complications during Intensive Care Unit (ICU) stay, catecholamine use (dose and duration of treatment), duration of mechanical ventilation, duration of VA-ECMO treatment, rate of rewarming, left ventricular ejection fraction (LVEF) on discharge and survival rate. The clinical characteristics of the group are presented in Table 1.

**Table 1:** Characteristics and outcomes of the 13 patients in severe accidental hypothermia with cardiocirculatory instability

Sex: Male	10 (77%)
Age [years]	Me 60; IQR [56-66]
Urban-type hypothermia	13 (100%)
Distance of transport [km]	Me 14, IQR [7-82]
Type of Transport	
HEMS	2 (15%)
Ambulance	10 (77%)
ECMO team to the patient	1 (8%)
Tc on admission [°C]	25.8 ± 2.1 (20.7–29.0)
HR on admission [min^-1^]	53 ± 12.9 (40–80)
SBP [mmHg]	Me 70 (50–80)
DBP [mmHg]	Me 40 (30–60)
Heart rhythm on admission	
Sinus rhythm	8 (62%)
Atrial fibrillation	2 (15%)
Junctional rhythm	3 (23%)
Complications during ICU stay	
Aspiration Pneumonia	1 (8%)
Lower Limb Ischemia	3 (23%)
Bleeding from Upper GI Tract	3 (23%)
Alcohol Withdrawal Syndrome	4 (31%)
Kidney Failure	3 (23%)
Cathecholamine use	
Adrenaline	4 (31%; mean dose 0.16 ug/kg/min)
Noradrenaline	9 (69%; mean dose 1.7 ug/kg/min)
Dopamine	2 (15%; mean dose 15 ug/kg/min)
Dobutamine	3 (23%; mean dose 16.7 ug/kg/min)
More than 1 cathecholamine used	5 (38%)
Cathecholamine administration [days]	Me 2 IQR [1–7]
Duration of mechanical ventilation [days]	Me 2 IQR [1,3–7,9]
Duration of VA-ECMO [hours]	Me 23 IQR [19–32]
Rate of rewarming [C/hrs.]	Me 1,8 IQR [1,5–3,0]
Duration of stay in the ICU [days]	Me 3 IQR [2,0–9,0]
LVEF on discharge [%]	56 ± 8 (40–65)
Survived	9 (69%)

M denotes mean value; *Me* median, *IQR* interquartile interval

Comparison of clinical parameters between survivors and non-survivors included core temperature (Tc), blood pressure and end-tidal carbon dioxide (EtCO_2_) at admission, as well as parameters of acid-base balance (pH, pCO_2_, pO_2_, base deficit), lactate level and clearance, serum glucose, electrolytes and creatinine, levels of creatine kinase, creatine kinase-MB and high sensivity troponin T (hsTnT), hemoglobin level and coagulation profile.

Blood samples were collected for the blood glucose and chemistry analysis at the time of admission and according to accepted schedule. Arterial blood gas analysis according to alpha-stat strategy (corrected to the normal body temperature) was assessed on admission and on 2, 4, 6, 8, 10, 12, 16, 20, 24 h of ICU stay. Blood tests were assayed by routine automated laboratory techniques (Cobas System 600, Roche Diagnostics GmbH, Manheim, Germany). All biochemical analyses were performed in the central hospital laboratory, certified with a cardiac and clinical chemistry program by RIQAS (Randox Quality Assessment Scheme, UK). Lactate clearance was calculated as followed [10]: $$ \begin{array}{l}\left[\left({\mathrm{lactate}}_{\mathrm{initial}} - {\mathrm{lactate}}_{\mathrm{delayed}}\right)/{\mathrm{lactate}}_{\mathrm{initial}}\right]\times \left.100\%\right].\\ {}\end{array} $$

Due to a small sample included in the study, the statistical analysis has been limited to presentation of descriptive statistics. Categorical variables were presented as numbers of subjects and percentages. Continuous variables were analyzed for normal distribution using the Shapiro–Wilk test and were presented as mean plus standard deviation and minimum and maximum for normal distribution and were presented as median values (Me) with lower and upper quartiles (IQR) for non-normal distribution.

## Results

**Fig. 1:**
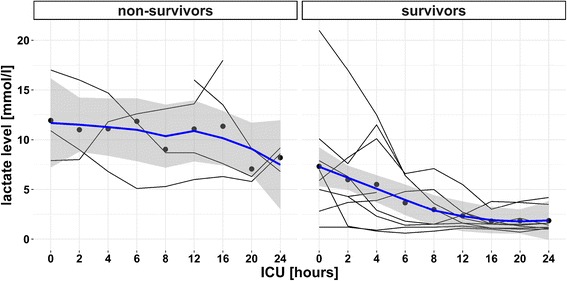
Individual patient lactate level profiles in first 24 h of ICU stay. Dots represent mean value of lactate level in all patients arterial blood sample for the following hours. Bold lines represent smooth curve fitted by loess function with 95% confidence interval

The comparison of clinical parameters and outcomes of survivors and non-survivors are presented in Table 2. Patients who died were older, with lower blood pressure and biochemical parameters abnormalities reflecting severe organ hypoperfusion, including significantly increased potassium level and more than doubled creatinine concentrations. The clinical characteristics of non-survivors, including time and cause of death are presented in Table 3. In survivors, arterial blood gas parameters (pH, BE, HCO_3_^-^) and lactates normalized faster. Lactate clearance in the first 24 h of ICU admission is presented in Fig. 1. There is also a considerable difference between end-tidal CO_2_ and arterial blood gas CO_2_ partial pressure on admission of about 20 mmHg (Table 2).

**Table 2:** Characteristics of survivors and non-survivors

Parameter	Survivors (*n* = 9)	Non-survivors (*n* = 4)
Age [years]	Me 57 IQR [55–60]	Me 73 IQR [64.5–82.5]
Tc at hospital admission [°C]	26.07 ± 1.51	25.32 ± 3.24
SBP at hospital admission [mmHg]	74.4 ± 8.82	57.5 ± 5
DBP at hospital admission [mmHg]	47.2 ± 7.55	35 ± 5.77
pH at hospital admission	7.14 ± 0.08	7.02 ± 0.15
pH after achieving normothermia	Me 7.32 IQR [7.32–7.33]	Me 7.23 IQR [7.03–7.32]
EtCO_2_ at hospital admission	17.3 ± 1.5	14.5 ± 2.5
PaCO_2_ at hospital admission [mmHg]	41.4 ± 8.2	26.4 ± 7.4
PaCO_2_ after achieving normothermia [mmHg]	Me 31.4 IQR [28.7–31.8]	Me 24.5 IQR [19.2–29.6]
PaO_2_ at hospital admission [mmHg]	232.5 ± 117.7	107.6 ± 50.7
Lactate level (mmol/l) at hospital admission	6.89 ± 5.94	12.57 ± 4.55
Lactate level (mmol/l) after achieving normothermia	Me 2.9 IQR [1.4–3.6]	Me 12.2 IQR [8.4–15.3]
Lactate clearance 6 h	46.9%	5.7%
Lactate clearance 24 h	73.0%	34.8%
Lactate clearance at normothermia	56.0%	5.7%
BE at hospital admission [mEq/L]	-14.322 ± 4.82	-23.45 ± 4.33
BE after achieving normothermia [mEq/L]	Me -9.2 IQR [-11.4 – -7.7]	Me -14 IQR [-22.1– -12.1]
HCO_3_ at hospital admission [mmol/L]	13.889 ± 4.09	7.225 ± 1.63
HCO_3_ after achieving normothermia [mmol/L]	16.389 ± 3.37	9.850 ± 4.78
K at hospital admission [mmol/L]	3.022 ± .76	4.975 ± .85
Na at hospital admission [mmol/L]	137.6 ± 6.4	143.2 ± 16.1
Glucose at hospital admission [mmol/L]	8.2 ± 2.97	6.1 ± 1.07
Creatinine at hospital admission [μmol/L]	Me 85 IQR [64–114]	Me 194 IQR [139-376]
CK at hospital admission [U/L]	Me 1374 IQR [1114–2397]	Me 4664 IQR [1724–18054]
CK 6 h [U/L]	Me 1195 IQR [495–4168]	Me 1948 IQR [595–6830]
CK-MB at hospital admission [U/L]	Me 115 IQR [100–132]	Me 278 IQR [164–394]
CK-MB 6 h [U/L]	Me 70 IQR [54–183]	Me 180 IQR [125-–249]
hsTnT at hospital admission [nmol/L]	Me 0.033 IQR [0.027–0.048]	Me 0.082 IQR [0.073–0.203]
hsTnT 6 h [nmol/L]	Me 0.064 IQR [0.038–0.372]	Me 0.097 IQR [0.091–0.17]
INR at hospital admission	1.46 ± .28	1.61 ± .23
APTT 0 h	37.7 ± 10.7	35.9 ± 15.1
PLT at hospital admission [10^3^/uL]	142 ± 66	124 ± 110
Hb at hospital admission [g/dl]	12.13 ± 4.0	10.90 ± 3.66
Hb after achieving normothermia [g/dl]	9.54 ± 1.94	9.15 ± 1.21

Continuous data presented with mean value with standard deviation (normal distribution) or as median with lower and upper quartiles (non-normal distribution). M denotes mean value; *Me* median, *IQR* interquartile interval

**Table 3:** The clinical characteristics of non-survivors

Sex/age (years)	Tc (°C)	VA ECMO treatment (hours)	Time of death (hours since admission)	Cause of death
M/66	20.7	42	216	gastrointestinal bleeding, hepatic failure, multiple organ failure
F/81	25.6	21	48	traumatic brain injury, gastrointestinal bleeding
M/63	27.0	26	36	methanol intoxication
F/84	28.0	16	20	gastrointestinal bleeding, renal insufficiency

After 6 h of therapy, normothermia was achieved in 12 patients (92.3%). None of the patients had been weaned off ECMO within the first 6 h of treatment. The outcome of 13 patients after 6 h of VA-ECMO treatment are presented in Table 4.

**Table 4:** Outcome of 13 patients after 6 hours of VA-ECMO treatment

Feature	Survivors	Non-survivors	Total
Normothermia	8/9 (88.9%)	4/4 (100%)	12/13 (92.3%)
Cathecholamine support (>1 drug)	7/9 (77.8%)	2/4 (50%)	9/13 (69.23%)
pH < 7.2	1/9 (11.1%)	2/4 (50%)	3/13 (23.07%)
Lack of lactate normalization	6/9 (66.7%)	4/4 (100%)	10/13 (76.92%)
Diuresis < 1 ml/kg/h	0/9 (0%)	2/4 (50%)	2/13 (15.4%)
Fluid resuscitation volume > 3000 ml/6 h	7/9 (77.8%)	4/4 (100%)	11/13 (84.6%)

## Discussion

In our study we have attempted to indicate unfavorable prognostic factors in a small, but uniform group of severely hypothermic patients with cardiocirculatory instability, treated with VA-ECMO. Those factors were: old age, low blood pressure on admission, low initial and post-warming PaCO_2_ partial pressure, low pH, large base deficit, high serum creatinine, potassium and lactate levels (marked at least six hours of rewarming). However, the study sample size is too small to assess any predictive value of individual factors related to survival. Thus, the main finding of the study is that most prominent feature which distinguishes survivors and non-survivors in our cohort are clinical and biochemical parameters reflecting organ hypoperfusion. At the moment the clinical criteria to be considered as the indications for ECLS rewarming are: failure to improve with external active and minimally invasive rewarming methods, life-threatening arrhythmia, hypotension (systolic blood pressure <90 mmHg), respiratory failure, refractory acidosis and comorbidities, which limit tolerance for the low-flow state of HT III [4]. Although our study was only observational in design and limited to small number of patients it indicates, that future studies on indications to extracorporeal rewarming in severely hypothermic, non-arrested patients should focus on the extent of hemodynamic disturbances. The mean systolic blood pressure in analyzed cohort was below 70 mmHg despite high catecholamine doses. Mair and Ruttmann proposed a scheme in which the criteria for introducing extracorporeal rewarming in non-arrested, severely hypothermic patients was SBP ≤60 mmHg and/or serious rhythm disorders [11]. Taking into account the results of our study and experience from SHTC, we were eager to accept those lower limits of blood pressure instead of current <90 mmHg. At the same time, in our opinion, there should be an emphasis on acid-base balance parameters, which – if they are not stabilized or normalized in the initial phase of emergency actions – indicate serious hemodynamic disturbances in the course of hypothermia.

Mortality rate until ICU discharge in our study was 31%. Vassal et al. in their study, which was also dedicated to urban-type hypothermia, reported the mortality rate of 38% with the core temperature (Tc) higher by 3 °C, mean systolic pressure higher by 16 mmHg, and all patients being rewarmed by non-invasive methods. Taking into account the study subgroup of patients with Tc < 29 °C (21 patients), the mortality was over 50% [12]. When comparing the results of both studies it could be concluded, that extracorporeal rewarming can be beneficial, at least in selected group of the patients. However despite numerous case reports, observation studies, rich clinical experience and expert guidelines, there is lack of randomized controlled trials and it is difficult to present convincing, evidence-based proofs of increased survival rate in patients treated by extracorporeal rewarming. In many cases, death is caused by concomitant, chronic conditions, trauma, and/or circumstances in which hypothermia occurred (such as asphyxia in drowning or avalanche burial) [4].

Ruttman et al. identified unfavorable prognostic factors in patients in cardiac arrest rewarmed with VA-ECMO. A single-factor analysis indicated elevated level of potassium, lower blood pH on admission, and preceding asphyxia as mortality factors [7]. In the whole analyzed group, about 75% of patients were asphyxiated due to drowning or avalanche burial. It is then possible, that the results of electrolyte and acid-base abnormalities did not reveal hypothermia per se, but were related to asphyxia. However, in our cohort hypothermia was induced by environmental exposure in urban setting in all cases, without episodes of asphyxia. Thus, the results of our preliminary study correspond with the results obtained by Ruttman et al.

An additional objective of this study was to evaluate the efficacy of the initial, 6-h period of VA-ECMO rewarming. The median duration of extracorporeal treatment in our group was 23 h and significantly exceeded the time required for achieving normothermia. Even within the group of survivors, after six hours of treatment, the patients’ circulatory parameters were not stable enough to safely wean-off VA-ECMO. The main goals of the ECLS are stabilization of the core temperature and hemodynamic status, including cerebral perfusion, particularly in patients after cardiac arrest [4, 11]. Prompt normalization of acid-base balance parameters during the first six hours of VA-EMCO treatment may be prognostically significant, and proves the treatment to be effective, but is not clinically exhaustive. Therefore, based on our data, it seems that the use of short-term extracorporeal treatment of hypothermia, although economically cost-effective, is clinically not appropriate.

Severely hypothermic patients with cardiocirculatory instability represent a vulnerable group of patients and it is important to evaluate treatment strategies with invasive techniques, as lack of randomized studies makes causal inference of the benefit of such strategies uncertain. However, our study has a number of limitations. The small sample size affects the reliability of the findings. There was no control group with which to compare changes in parameters analyzed. The implementation of a standardized approach makes the results from this cohort study more valid, but the findings need to be interpreted with caution.

## Conclusions

In our preliminary study more pronounced markers of cardiocirculatory instability and organ hypoperfusion were observed in non-survivors. Future studies on indications to extracorporeal rewarming in severely hypothermic, non-arrested patients should focus on the extent of hemodynamic disturbances. In our opinion, there should be an emphasis on acid-base balance parameters, which – if they are not stabilized or normalized in the initial phase of emergency actions – indicate serious hemodynamic disturbances in the course of hypothermia. Short term (<6 h) extracorporeal treatment in severe hypothermic, non-arrested patients seems to be not clinically appropriate.

## Abbreviations

APTT: Activated partial thromboplastin time
CK: Creatine kinase
CK-MB: Cardiac isoenzyme of creatine kinase
CO: Cardiac output
DBP: Diastolic blood pressure
ECLS: Extracorporeal life support
EtCO_2_: End-tidal CO_2_
Hb: Hemoglobin
hsTnT: High sensitivity troponin
ICU: Intensive Care Unit
INR: International normalized ratio
IQR: Lower and upper quartiles
LVEF: Left ventricular ejection fraction
Me: Median values
PaCO_2_: Partial pressure of CO_2_ in arterial blood
PaO_2_: Partial pressure of O_2_ in arterial blood
PLT: Platelets
SBP: Systolic blood pressure
SHTC: Severe Hypothermia Treatment Centre
Tc: Core body temperature
VA-ECMO: Extracorporeal membrane oxygenation arteriovenous configuration
